# Cancer-Associated Fibroblasts Affect Tumor Metabolism and Immune Microenvironment in Gastric Cancer and Identification of Its Characteristic Genes

**DOI:** 10.1155/2023/1424589

**Published:** 2023-01-30

**Authors:** Chanchan Gao, Fei Liu, Qiuju Ye, Aiping Guo

**Affiliations:** ^1^Department of Oncology, Zhongda Hospital, School of Medicine, Southeast University, Nanjing 210009, China; ^2^Department of Medical Oncology, Luhe People's Hospital of Nanjing, Nanjing 21009, China

## Abstract

**Background:**

Cancer-associated fibroblasts (CAFs) have reported widely involved in cancer progression. However, its underlying mechanism in gastric cancer is still not clarified.

**Methods:**

The data used in this study were all downloaded from the Cancer Genome Atlas database. R software and the R packages were used for all the analyses.

**Results:**

In our study, we first quantified the CAFs infiltration using the ssGSEA algorithm. The clinical correlation result showed that CAFs were associated with a worse prognosis and clinical features. Pathway enrichment also indicated several oncogenic pathways in GC patients with high CAFs infiltration, including epithelial-mesenchymal transition (EMT), myogenesis, allograft rejection, the inflammatory response, and IL2/STAT5 signaling. Furthermore, FNDC1 and RSPO3 were identified as the characteristic genes of CAFs through two machine learning algorithms, LASSO logistic regression and SVM-RFE. The following analysis showed that FNDC1 and RSPO3 were associated with more progressive clinical features and had a good prediction efficiency of the CAFs infiltration status in GC patients. Pathway enrichment and genomic instability were performed to explore the underlying mechanisms of FNDC1 and RSPO3. Immune infiltration analysis showed that CAFs were positively correlated with M2 macrophages. Moreover, we found that the GC patients with low CAFs infiltration were more sensitive to immunotherapy. Also, the CAFs, FNDC1, and RSPO3 could generate a certain effect on the sensitivity of doxorubicin, mitomycin, and paclitaxel.

**Conclusions:**

In summary, our study comprehensively investigated the role of CAFs in GC, which might be associated with immunotherapy sensitivity. Meanwhile, FNDC1 and RSPO3 were identified as the underlying targets of GC.

## 1. Introduction

Gastric cancer (GC) is the fifth most common cancer around the world, with over one million new cases diagnosed annually [1]. There has been a noticeable increase in the incidence of GC worldwide, along with its high mortality and metastasis rate [1]. At present, surgery is still the first-line therapy option for early-staged GC and can lead to persistent prognosis benefits [2]. Meanwhile, combined therapies, including chemotherapy and targeted therapy, have also prolonged the overall survival (OS) of advanced GC patients [3]. Despite this, however, the five years survival rate of advanced GC patients is still less than 20% [3]. Therefore, early diagnosis and precise therapy of GC patients remain the focus of research.

Tumor cells are continuously affected by the tumor microenvironment (TME) they exist in, the components of which mainly consist of immune and stromal cells [4]. Cancer-associated fibroblasts (CAFs) are one of the most prominent cell types in TME that can influence tumor progression in multiple manners [5]. CAFs can secrete specific biological factors such as EGF, TGF-*β*, and IL6 to facilitate tumor malignant phenotype, including tumor neovascularization and immune escape, leading to tumor deterioration [6]. Meanwhile, CAFs can regulate tumor metabolism. CAFs can enhance glycolysis and excrete plenty of lactic acid and hydrogen ions, forming an acidic microenvironment to inhibit the activity of immune cells. Also, the metabolites of lactic acid and pyruvate produced by CAFs can be used as nutrients for tumor cells to stimulate their growth [7]. Recently, increasing attention has been paid to the role of CAFs in cancers for its diverse biological functions. For instance, Liubomirski et al. found that in breast cancer, the interactions between cancer cells and CAFs can significantly enhance the prometastatic phenotypes of the TME, further resulting in the higher angiogenesis, migratory, and invasive potential of cancer cells [8]. In esophageal squamous cell carcinoma, Jolly et al. revealed that CAFs can secrete IL-6 and exosomal miR-21 to induce the generation of monocytic myeloid-derived suppressor cells, which not only suppressed immune function but also enhanced drug resistance [9]. However, few studies have focused on the role of CAFs in GC, and therefore, it is meaningful to explore the underlying effect of CAFs to guide the treatment of GC.

Advancements in bioinformatic analysis provide a great convenience for researchers in investigating the underlying biological mechanisms of diseases [10]. In our study, we quantified the CAFs infiltration using the ssGSEA algorithm and comprehensively explored its role in GC. CFNDC1 and RSPO3 were identified as the characteristic genes of CAFs through two machine learning algorithms, LASSO logistic regression and SVM-RFE. Further following analysis showed that FNDC1 and RSPO3 were associated with more progressive clinical features and had a good prediction efficiency of the CAFs infiltration status in GC patients. Pathway enrichment and genomic instability were performed to explore the underlying mechanisms of FNDC1 and RSPO3. Immune infiltration analysis showed that CAFs were positively correlated with M2 macrophages. Moreover, we found that the GC patients with low CAFs infiltration were more sensitive to immunotherapy. Also, the CAFs, FNDC1, and RSPO3 could generate a certain effect on the sensitivity of doxorubicin, mitomycin, and paclitaxel.

## 2. Methods

### 2.1. Available Data Acquisition

The public transcription profiles and clinical information of GC patients were downloaded from The Cancer Genome Atlas database-TCGA-STAD project. The expression profile was in TPM form and was annotated based on the Homo sapiens.GRCh38.107.gtf file. Clinical information was in a “bcr-xml” file and extracted using the Perl code. Differentially expressed genes (DEGs) analysis was performed using the limma package with the threshold of |logFC| > 1 and adj.*P* < 0.05. The basic information of enrolled patients is shown in Table 1.

### 2.2. Single Sample Gene Set Enrichment Analysis

Single sample gene set enrichment analysis (ssGSEA) was used to quantify the relative enrichment score of CAFs [11]. The genes used for quantification were ACTA2, FAP, PDGFRB, CAV1, PDPN, PDGFRA, ZEB1, FOXF1, SPARC, MMP2, and FN1 from the CellMarker website (https://bio-bigdata.hrbmu.edu.cn/CellMarker/). The metabolism and immune-related pathways were also quantified using ssGSEA analysis.

### 2.3. Pathway Enrichment Analysis

Pathway enrichment analysis was performed using the gene set enrichment analysis (GSEA) algorithm, and the analyzed gene set was the Hallmark signature. The terms with |normalized enrichment score (NES)| > 1 and adj.*P* < 0.05 were considered statistically significant.

### 2.4. Characteristic Gene Identification

Two machine learning algorithms, LASSO logistic regression and support vector machine recursive feature elimination (SVM-RFE), were utilized to identify the characteristic genes of specific features [12]. Receiver operating characteristic (ROC) curves were used to evaluate the prediction efficiency of characteristic genes. Principal component analysis (PCA) was performed using the ade4 package in R environments.

### 2.5. Immune Infiltration and Genomic Analyses

The quantification of the immune microenvironment of GC was conducted using the CIBERSORT algorithm, and 22 types of infiltrating immune cells were extracted [13]. The scores of TMB and MSI were downloaded from the TCGA database. The tumor stemness index mRNAsi and EREG-mRNAsi were calculated according to the one-class logistic regression (OCLR) machine learning algorithm of the previous study [14].

### 2.6. Immunotherapy and Drug Sensitivity Analyses

Tumor Immune Dysfunction and Exclusion (TIDE) analysis (https://tide.dfci.harvard.edu/) and submap algorithm were utilized to evaluate the immunotherapy response rate of GC patients. Drug sensitivity analysis was conducted based on the data from the Genomics of Drug Sensitivity in Cancer (GDSC) database (https://www.cancerrxgene.org).

### 2.7. Statistical Analysis

R software was responsible for all the analysis. Here, the comparison with *P* value less than 0.05 was considered statistically significant. The ggplot2 package was utilized for most plots [15]. The correlation of continuous variables was compared using the Spearman method. The comparison of variables with a normal distribution was performed using the Student's *T*-test. Kaplan–Meier (KM) survival curves were used to evaluate the prognosis effect of specific index.

## 3. Results

### 3.1. Quantification of CAFs in TCGA Data

**Figure 1:**
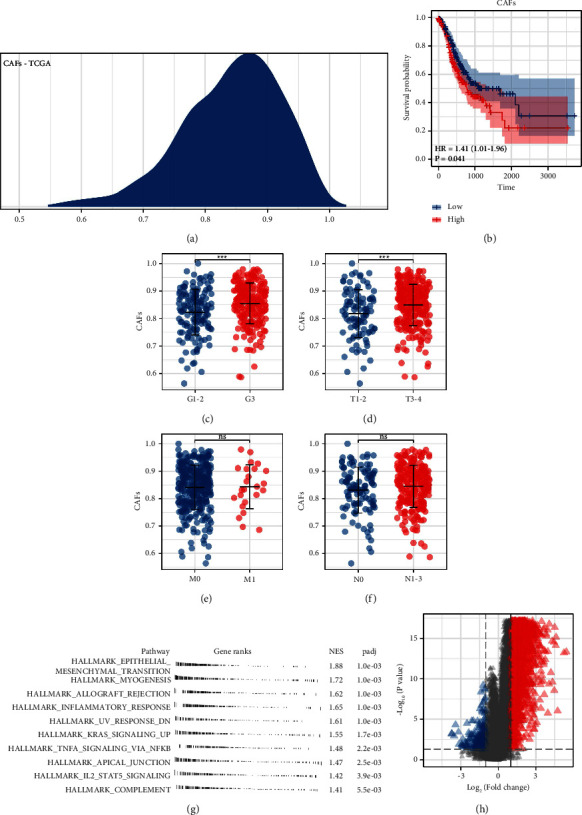
Exploration of CAFs in GC. (a) ssGSEA was performed to quantify the relative content of CAFs in TCGA database. (b) KM survival curves showed that CAFs were associated with a worse prognosis. (c–f) The difference of CAFs infiltration in patients with different clinical features. (g) Pathway enrichment analysis of CAFs. (h) DEGs analysis between high and low CAFs infiltration with the threshold of |logFC| > 1 and adj.*P* < 0.05.

The flowchart of whole study is shown in Figure S1. First, based on the marker genes mentioned above, the relative infiltration of CAFs in GC tissue was quantified using the ssGSEA algorithm (Figure 1(a)). KM survival curve showed that the patients with higher CAFs infiltration might have a worse overall survival (OS) (Figure 1(b), HR = 1.41, *P*=0.041). Furthermore, we explored the CAFs differences in patients with different clinical features. The result showed that CAFs might be associated with a more progressive grade and *T* stage (Figures 1(c) and 1(d)). However, no significant difference was observed in *M* and *N* stages (Figures 1(e) and 1(f)). Pathway enrichment analysis showed that in the patients with higher CAFs infiltration, the pathway of epithelial-mesenchymal transition (EMT), myogenesis, allograft rejection, inflammatory response, and IL2/STAT5 signaling were remarkably enriched in (Figure 1(g)).

### 3.2. Identification of the Characteristic Genes of CAFs

**Figure 2:**
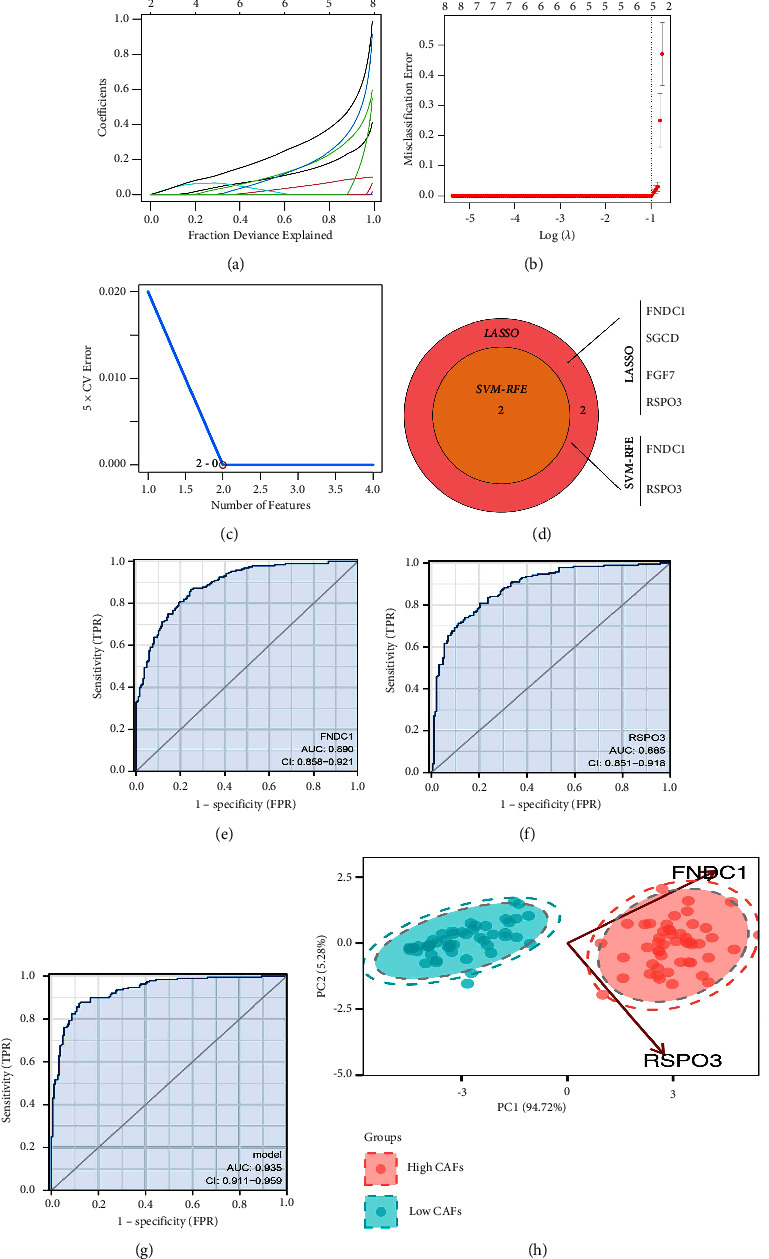
Identification of the characteristic genes of CAFs. (a, b) LASSO logistic regression was used to identify the characteristic genes of CAFs. (c) SVM-RFE was used to identify the characteristic genes of CAFs. (d) FNDC1 and RSPOS were identified as the characteristic genes of CAFs. (e, f) ROC curves to evaluate the prediction efficiency of FNDC1 and RSPOS on CAFs infiltration status. (g) A logistic regression was performed based on the FNDC1 and RSPO3. The formula was “score = −95.2708 + 15.9714 *∗* FNDC1 + 13.5927 *∗* RSPO3.” (h) PCA analysis of FNDC1 and RSPO3 in different CAFs infiltration patients.

Then, we performed the DEGs analysis with the threshold of |logFC| > 1 and adj.*P* < 0.05. A total of 268 downregulated and 1697 upregulated DEGs were identified (Figure 1(h)). LASSO logistic regression and the SVM-RFE algorithm were used to identify the characteristic genes of CAFs (Figures 2(a)–2(c)). LASSO logistic regression identified four genes, including FNDC1, SGCD, FGF7, and RSPO3. Further, among these four genes, the SVM-RFE algorithm screened two genes FNDC1 and RSPOS, as the characteristic genes of CAFs (Figure 2(d)). ROC curves showed that FNDC1 and RSPO3 had great prediction in the CAFs infiltration status of GC patients (Figures 2(e) and 2(f), FNDC1, AUC = 0.890; RSPO3, AUC = 0.885). Then, logistic regression was performed based on the FNDC1 and RSPO3. The formula was “score = −6.691 + 0.9797 *∗* FNDC1 + 1.2415 *∗* RSPO3.” The ROC curve showed that the logistic score had an excellent prediction ability of the CAFs infiltration of GC patients (Figure 2(g)). PCA analysis indicated that the genes FNDC1 and RSPO3 could effectively distinguish the GC patients with high and low CAFs infiltration (Figure 2(h)).

### 3.3. Prognosis Effect and Clinical Correlation of FNDC1 and RSPO3

**Figure 3:**
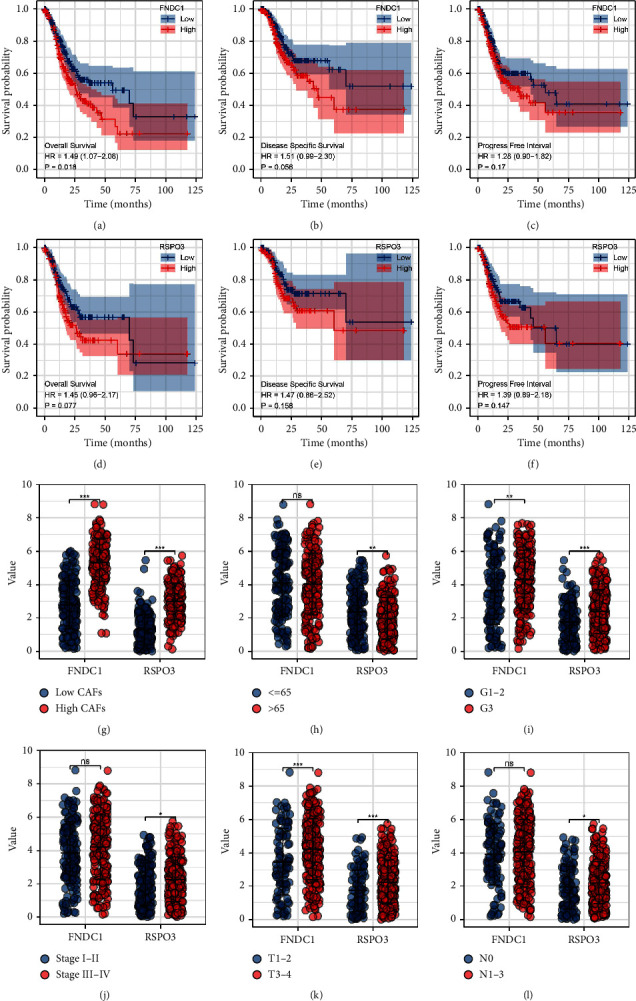
Prognosis effect and clinical correlation of FNDC1 and RSPOS. (a–c) The OS, DSS, and PFI of FNDC1 in TCGA database. (d–f) The OS, DSS, and PFI of RSPO3 in the TCGA database. (g–l) The expression level of FNDC1 and RSPOS in different GC patients.

KM survival curves showed that the patients with high FNDC1 and RSPO3 expression might have a worse OS, DSS and PFI (Figures 3(a)–3(f)). Also, we found that the patients with higher CAFs infiltration might have a higher FNDC1 and RSPO3 expression (Figure 3(g)). Meanwhile, the young patients (≤65 years old) tend to have a higher RSPO3 expression (Figure 3(h)); the G3 GC patients might have a higher FNDC1 and RSPO3 expression than G1-2 patients (Figure 3(i)); the stage III-IV patients might have an higher RSPO3 expression (Figure 3(j)); the T3-4 GC patients might have a higher FNDC1 and RSPO3 expression than T1-2 patients (Figure 3(k)); the N1-3 GC patients might have a higher RSPO3 expression than N0 patients (Figure 3(l)).

### 3.4. Biological Explorations of FNDC1 and RSPO3

**Figure 4:**
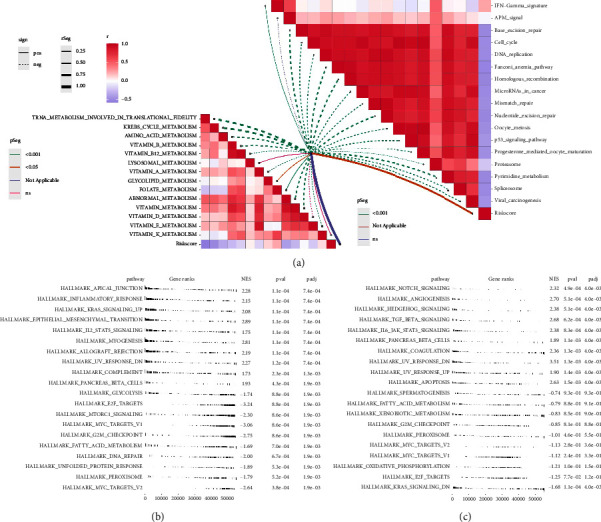
Pathway enrichment analyses of FNDC1 and RSPOS. (a) Pathway enrichment analysis of FNDC1 using the GSEA analysis. (b) Pathway enrichment analysis of RSPO3 using the GSEA analysis. (c) Pathway enrichment analysis of FNDC1 using the GSEA analysis.

**Figure 5:**
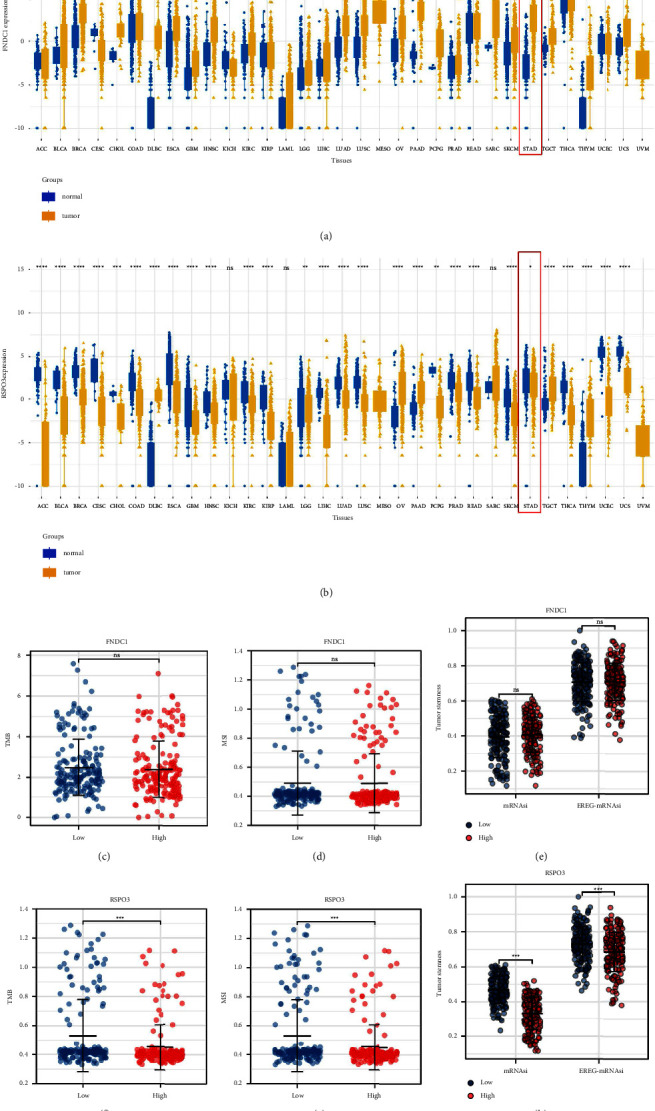
Further exploration of FNDC1 and RSPOS. (a, b) Pan-cancer analysis illustrates the expression pattern of FNDC1 and RSPOS. (c–e) The correlation between FNDC1 and TMB, MSI, and tumor stemness index. (f–h) The correlation between RSPO3 and TMB, MSI, and tumor stemness index.

CAFs have been reported to affect tumor metabolism. Pathway correlation analysis indicated that CAFs was negatively correlated with TRN-*α* metabolism, KREBS cycle metabolism, amino acid metabolism, vitamin metabolism, abnormal metabolism, and vitamin metabolism, yet positively correlated with folate metabolism (Figure 4(a)). We next explored the underlying pathways of FNDC1 and RSPO3. Pathway enrichment analysis of RSPO3 showed that the pathway of the apical junction, inflammatory response, KRAS signaling, and EMT were significantly enriched in the patients with high RSPO3 expression (Figure 4(b)). For FNDC1, the pathway of NOTCH signaling, angiogenesis, hedgehog signaling, TGF-*β* signaling, and IL6/JAK/STAT3 signaling were significantly enriched in (Figure 4(c)). Pan-cancer analysis revealed the expression patterns of FNDC1 and RSPO3 in solid cancers. The result showed that FNDC1 was upregulated, while RSPO3 was downregulated in GC tissue (Figures 5(a) and 5(b)). Genomic instability analysis showed that FNDC1 had no significant effect on TMB, MSI, and tumor stemness index (Figures 5(c)–5(e)). However, RSPO3 might be associated with a lower TMB, MSI and stemness index (Figures 5(f)–5(h)). Immune analysis showed that FNDC1 was positively correlated with NK cells, macrophages, and iDC, while negatively correlated with Th17 cells (Figure S2A); RSPO3 was positively correlated with NK cells, mast cells, and pDC yet negatively correlated with Th17 cells and Th2 cells (Figure S2B).

### 3.5. CAFs Is Positively Correlated with M2 Macrophages

**Figure 6:**
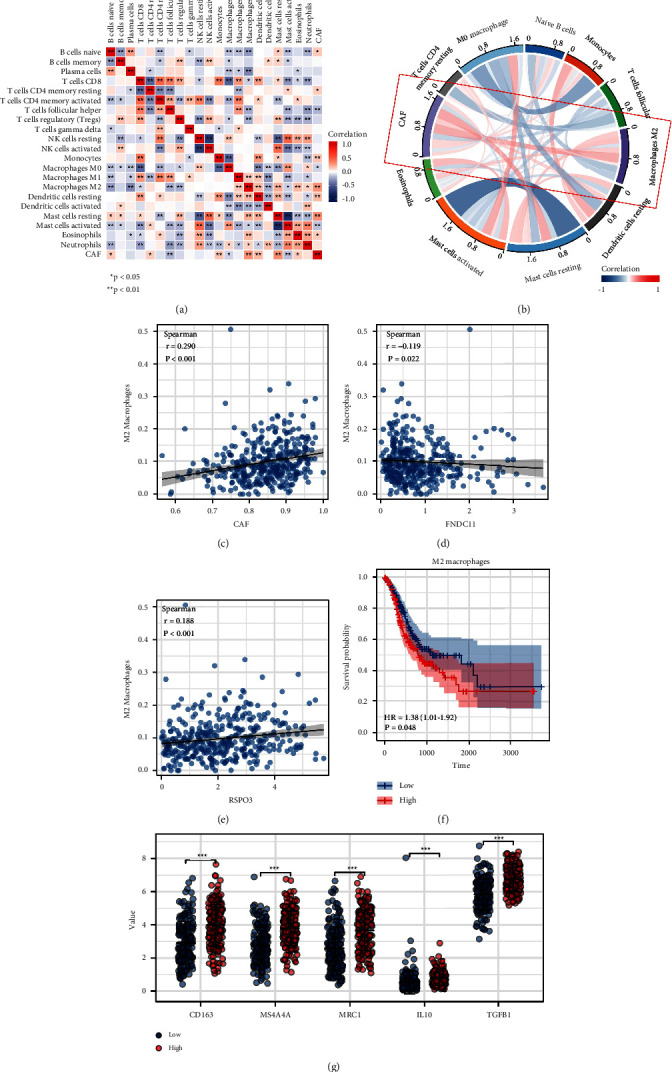
Immune infiltration analysis. (a) The correlation of CAFs and the quantified immune cells. (b) CAFs were positively correlated with M2 macrophages. (c) The correlation between M2 macrophages and CAFs, FNDC1, and RSPO3. (d) KM survival curves of the M2 macrophages. (e) The characteristic makers and factors were all highly expressed in the samples with high CAFs infiltration. (f) The KM survival curve showed that M2 macrophages might be associated with a poor prognosis. (g) The characteristic makers and factors were all highly expressed in the samples with high CAFs infiltration.

The crosstalk between different cells can significantly affect the TME of GC. The CIBERSORT algorithm was used for immune cell infiltration. The correlation of CAFs and the quantified immune cells are shown in Figure 6(a). The result showed that CAFs was positively correlated with naïve B cells, resting CD4+ memory T cells, monocytes, M2 macrophages, resting dendritic cells, resting mast cells, and eosinophils, yet negatively correlated with follicular helper T cells, M0 macrophages, and activated mast cells (Figures 6(b) and 6(c)). Moreover, we found that FDNC1 was negatively, while RSPO3 was positively correlated with M2 macrophages (Figures 6(d) and 6(e)). Also, the KM survival curve showed that M2 macrophages might be associated with a poor prognosis (Figure 6(f)). Meanwhile, the characteristic makers and factors were all highly expressed in the samples with high CAFs infiltration (Figure 6(g)).

### 3.6. CAFs and Its Characteristic Genes Were Associated with the Sensitivity of Immunotherapy and Chemotherapy

**Figure 7:**
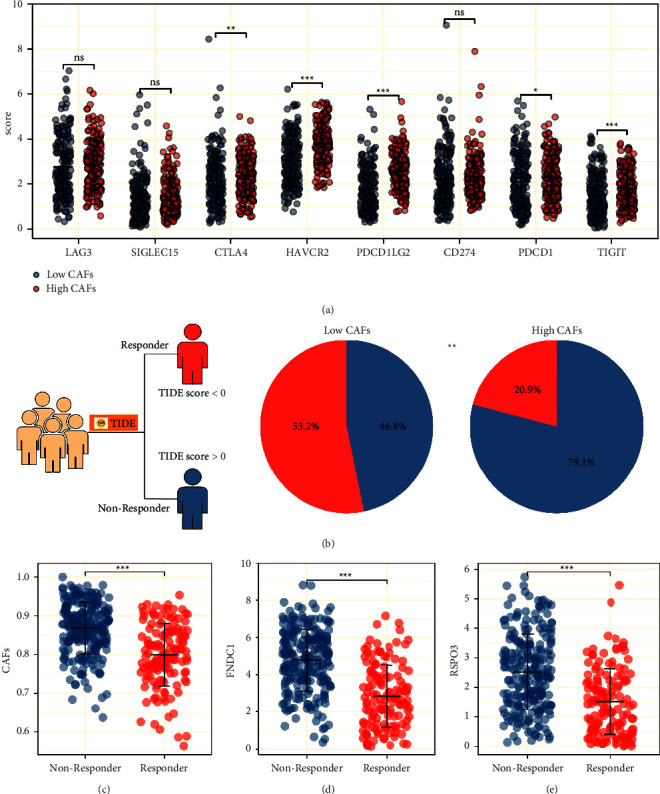
CAFs can affect the immunotherapy response of GC. (a) Multiple immune checkpoints level in GC patients with high and low CAFs infiltration. (b) TIDE analysis was performed to evaluate the immunotherapy response difference between high and low CAFs infiltration patients. (c–e) The difference of CAFs, FNDC1, and RSPO3 in immunotherapy responders and nonresponders patients.

**Figure 8:**
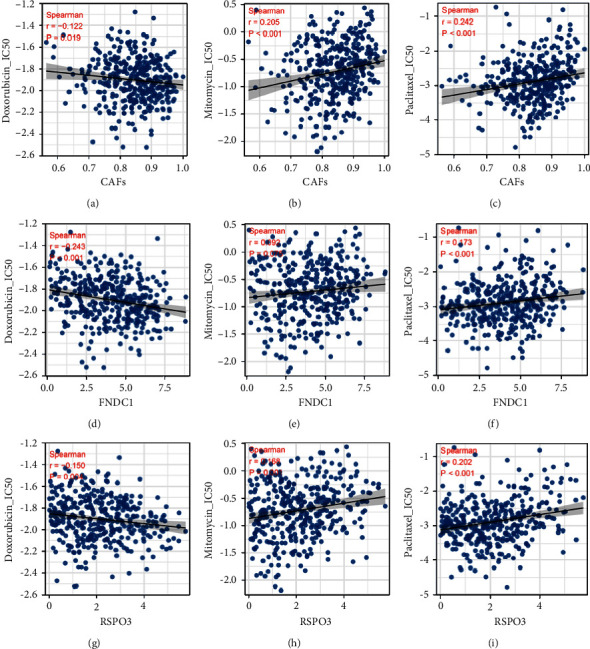
Drug sensitivity analysis. (a–c) The correlation of CAFs and the IC_50_ of doxorubicin, mitomycin, and paclitaxel. (d–f) The correlation of FNDC1 and the IC_50_ of doxorubicin, mitomycin, and paclitaxel. (g–i) The correlation of RSPO3 and the IC_50_ of doxorubicin, mitomycin, and paclitaxel.

Immunotherapy is a novel therapeutic option for advanced GC. Thus, we explored the underlying difference in immunotherapy sensibility between high and low CAFs infiltration patients. Immune checkpoint correlation analysis showed that CTLA4, HAVCR2, PDCD1LG2, PDCD1, and TIGIT were differentially expressed in high and low CAFs infiltration patients (Figure 7(a)). The TIDE analysis was then performed, in which the patients with TIDE a score >0 were defined as nonresponders and <0 were defined as responders. The result showed in low CAFs infiltration patients, the proportion of immunotherapy responders was 53.2%. However, in high CAFs infiltration patients, the proportion of immunotherapy responders was only 20.9%, indicating that low CAFs infiltration GC patients might be more sensitive to immunotherapy (Figure 7(b)). Submap analysis indicated that the patiens with low CAFs infiltration might be more sensitive to both PD-1 and CTLA4 therapies (Figure S3). Considering the significant correlation between CAFs and M2 macrophages, we further explored the effect of M2 macrophages on immunotherapy. Results showed a positive correlation between the TIDE score and M2 macrophages (Figure S4A). Moreover, we found that the patients with high M2 macrophages infiltration tend to have a higher TIDE score, as well as a lower percentage of immunotherapy responders (Figures S4B and S4C). Moreover, the immunotherapy responder had a low CAFs level (Figure 7(c)), as well as a lower FNDC1 and RSPO3 expression (Figures 7(d) and 7(e)). Drug sensitivity analysis showed that CAFs were negatively correlated with the IC_50_ of doxorubicin, while positively correlated with the IC_50_ of mitomycin and paclitaxel (Figures 8(a)–8(c)); FNDC1 was negatively correlated with the IC_50_ of doxorubicin, while positively correlated with the IC_50_ of paclitaxel (Figures 8(d)–8(f)); RSPO3 was negatively correlated with the IC_50_ of doxorubicin, while positively correlated with the IC_50_ of mitomycin and paclitaxel (Figures 8(g)–8(i)).

## 4. Discussion

A common cancer, GC poses one of the most serious public health problems [1]. CAFs are an important part of the TME in GC that can significantly affect cancer progression. Therefore, a deep investigation of CAFs and their related molecule targets would contribute to understanding the intrinsic biological mechanism of GC. In medical research, the investigation and analysis of the classification or prediction of response variables in biomedical research are often challenging due to the data sparsity generated by limited sample sizes and a moderate or very large number of predictors. Bioinformatic analysis can effectively solve this contradiction and is a powerful tool for screening clinical predictors [16].

In our study, we first quantified the CAFs infiltration using the ssGSEA algorithm. The clinical correlation result showed that CAFs were associated with a worse prognosis and clinical features. Pathway enrichment also indicated several oncogenic pathways in GC patients with high CAFs infiltration. Further, FNDC1 and RSPO3 were identified as the characteristic genes of CAFs through two machine learning algorithms, LASSO logistic regression and SVM-RFE. The following analysis showed that FNDC1 and RSPO3 were associated with more progressive clinical features and had a good prediction efficiency of the CAFs infiltration status in GC patients. Pathway enrichment and genomic instability were performed to explore the underlying mechanisms of FNDC1 and RSPO3. Immune infiltration analysis showed that CAFs were positively correlated with M2 macrophages. Moreover, we found that the GC patients with low CAFs infiltration were more sensitive to immunotherapy. Also, the CAFs, FNDC1, and RSPO3 could generate a certain effect on the sensitivity of doxorubicin, mitomycin, and paclitaxel.

Generally, in TME, the content of CAF is the most abundant, and it can affect the occurrence and development of cancer through intercellular contact, the release of various regulatory factors, and the remodeling of the extracellular matrix [17]. In colon cancer, Hu et al. indicated that CAFs could secret the exosome miR-92a-3p that was engulfed by colon cancer cells, further activating Wnt/*β*-catenin pathway and inhibiting mitochondrial apoptosis, leading to metastasis and chemotherapy resistance [18]. Su et al. revealed that CD10^+^ GPR77^+^ CAFs could induce cancer formation and chemoresistance through sustaining tumor stemness [19]. Wen et al. indicated that CAFs-derived IL32 could promote breast cancer cell invasion and metastasis through integrin *β*3-p38 MAPK signaling [20]. Pathway enrichment analysis showed that CAFs could activate the EMT, KRAS, and IL2/STAT5 signaling. In GC, Li et al. found that cancer-associated neutrophils could induce EMT through IL-17a to facilitate the invasion and migration of cancer cells [21]. Also, Wang et al. indicated that the downregulation of miRNA-214 in CAFs could enhance the migration and invasion of GC cells by targeting FGF9 and inducing EMT [22]. Our results were consistent with previous studies, which reflect the validity of the analysis.

Through machine learning algorithms, FNDC1 and RSPO3 were identified as the characteristic genes of CAFs. FNDC1, whose full name is “fibronectin type III domain containing 1”, has been reported to promote GC development. Jiang et al. demonstrated that FNDC1 could facilitate the invasion of GC by regulating the Wnt/*β*-catenin signaling and is correlated with peritoneal metastasis [23]. RSPO3 has been reported as being widely involved in cancer progression. For example, Chen et al. revealed that RSPO3 could enhance the aggressiveness of bladder cancer through Wnt/*β*-catenin and Hedgehog signaling pathways [24]. Fischer et al. found that in colon cancer with Wnt mutations, RSPO3 antagonism could hamper the malignant biological behavior of cancer cells [25]. However, virtually no study explored the RSPO3 in GC. Our study comprehensively investigated the underlying role of RSPO3 in GC, which can provide direction for future studies. In clinical practice, detecting the relative expression levels of FNDC1 and RSPO3 could indicate the CAFs infiltration level of patients, as well as their response on GC immunotherapy.

Interestingly, immune infiltration analysis showed that CAFs were associated with M2 macrophages. The interaction between different cells can significantly affect the remodeling effects of TME [26]. Previous studies have shown the underlying crosstalk between CAFs and M2 macrophages. Based on a coculture system, Cho et al. found that cancer-stimulated CAFs could promote M2 macrophage activation through secreting IL6 and GM-CSF [27]. Meanwhile, from a review summarized by Gunaydin, the interaction between CAFs and tumor-associated macrophages in TME can enhance tumorigenesis and immune escape [28]. Notably, our results also showed that in patients with low CAFs infiltration, the response rate to immunotherapy is higher (53.2% vs. 23.9%). Immunotherapy has shown a promising effect for specific advanced GC patients.

Although our research is based on high-quality bioinformatics analysis, some limitations should be noticed. First, the potential race bias is hard to ignore. Most patients enrolled in our study were from Western populations, which might decrease the credibility of our conclusions. Second, detailed laboratory examinations are hard to obtain. If all the data from all examinations can be obtained, our conclusion will be more abundant.

## Figures and Tables

**Table 1:** Basic information of enrolled patients.

Features		Numbers (*n*)	Percentage (%)
Age	≤65	197	44.5
	>65	241	54.4
	Unknown	5	1.1

Gender	Female	158	35.7
	Male	285	64.3

Grade	G1	12	2.7
	G2	159	35.9
	G3	263	59.4
	Unknown	9	2.0

Stage	Stage I	59	13.3
	Stage II	130	29.3
	Stage III	183	41.3
	Stage IV	44	9.9
	Unknown	27	6.1

T stage	T1	23	5.2
	T2	93	20.9
	T3	198	44.7
	T4	119	26.9
	Unknown	10	2.3

M stage	M0	391	88.3
	M1	30	6.8
	Unknown	22	4.9

N stage	N0	132	29.8
	N1	119	26.9
	N2	85	19.2
	N3	88	19.9
	Unknown	19	4.3

## Data Availability

The raw data mentioned in this study can be downloaded from online databases. The data used to support the findings of this study are available from the corresponding author upon request.

## Supplementary Materials

Supplementary Materials Figure S1: the flowchart of whole study. Figure S2: immune correlation analysis of FNDC1 and RSPO3. Notes: A: immune correlation analysis of FNDC1; B: immune correlation analysis of RSPO3. Figure S3: submap analysis was used to indicate patients' sensitivity to PD-1 and CTLA4 therapy. Figure S4: effect of M2 macrophages on GC immunotherapy. Notes: A: correlation of M2 macrophages and TIDE score; B: TIDE score in patients with high and low M2 macrophages infiltration; C: the percentage of immunotherapy responders and nonresponders in patients with high and low M2 macrophages infiltration.
