# Supplementary figures and images for: Cancer-Associated Fibroblasts Affect Tumor Metabolism and Immune Microenvironment in Gastric Cancer and Identification of Its Characteristic Genes
Source: J Oncol. 2023 Jan 30;2023:1424589. doi: 10.1155/2023/1424589 (PMC9902124; doi:10.1155/2023/1424589)

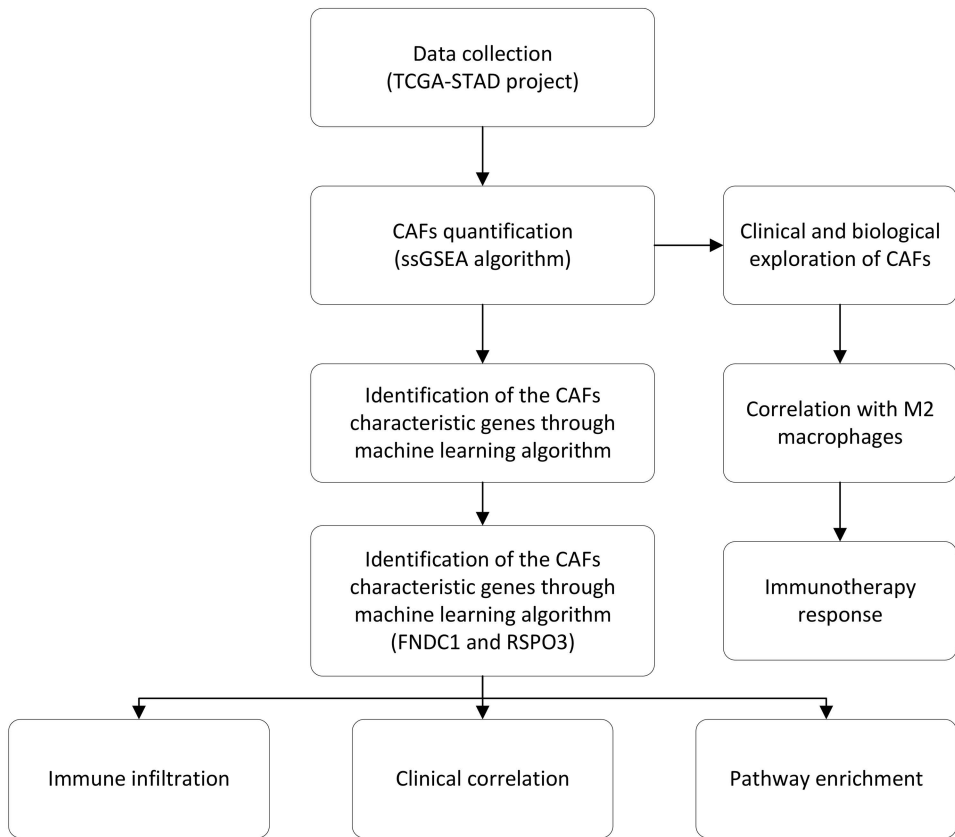

Supplement: Supplementary Materials — Figure S1: the flowchart of whole study. Figure S2: immune correlation analysis of FNDC1 and RSPO3. Notes: A: immune correlation analysis of FNDC1; B: immune correlation analysis of RSPO3. Figure S3: submap analysis was used to indicate patients' sensitivity to PD-1 and CTLA4 therapy. Figure S4: effect of M2 macrophages on GC immunotherapy. Notes: A: correlation of M2 macrophages and TIDE score; B: TIDE score in patients with high and low M2 macrophages infiltration; C: the percentage of immunotherapy responders and nonresponders in patients with high and low M2 macrophages infiltration. [file 1424589.f1.zip › Figure S1 (1).pdf]

**A**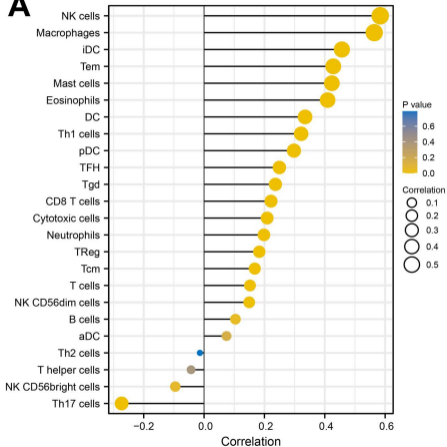**B**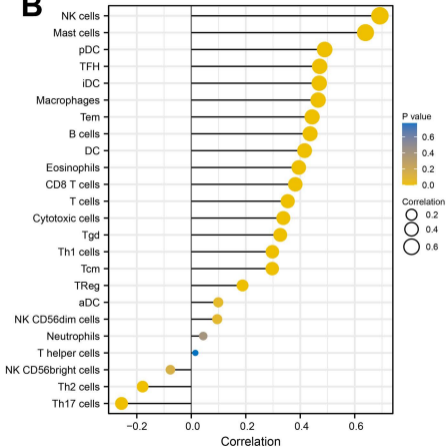

Supplement: Supplementary Materials — Figure S1: the flowchart of whole study. Figure S2: immune correlation analysis of FNDC1 and RSPO3. Notes: A: immune correlation analysis of FNDC1; B: immune correlation analysis of RSPO3. Figure S3: submap analysis was used to indicate patients' sensitivity to PD-1 and CTLA4 therapy. Figure S4: effect of M2 macrophages on GC immunotherapy. Notes: A: correlation of M2 macrophages and TIDE score; B: TIDE score in patients with high and low M2 macrophages infiltration; C: the percentage of immunotherapy responders and nonresponders in patients with high and low M2 macrophages infiltration. [file 1424589.f1.zip › Figure S2 (1).pdf]

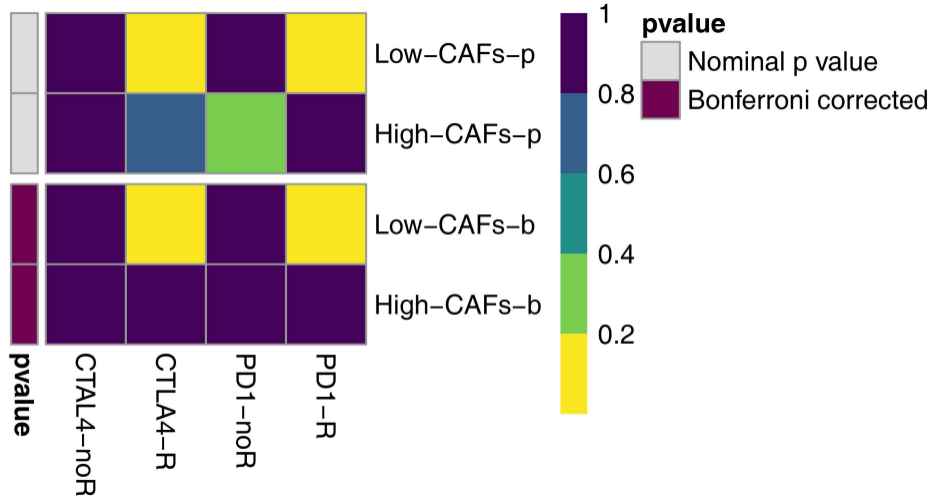

Supplement: Supplementary Materials — Figure S1: the flowchart of whole study. Figure S2: immune correlation analysis of FNDC1 and RSPO3. Notes: A: immune correlation analysis of FNDC1; B: immune correlation analysis of RSPO3. Figure S3: submap analysis was used to indicate patients' sensitivity to PD-1 and CTLA4 therapy. Figure S4: effect of M2 macrophages on GC immunotherapy. Notes: A: correlation of M2 macrophages and TIDE score; B: TIDE score in patients with high and low M2 macrophages infiltration; C: the percentage of immunotherapy responders and nonresponders in patients with high and low M2 macrophages infiltration. [file 1424589.f1.zip › Figure S3 (1).pdf]

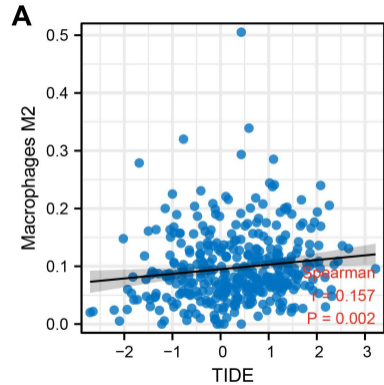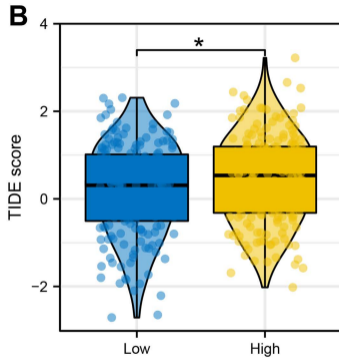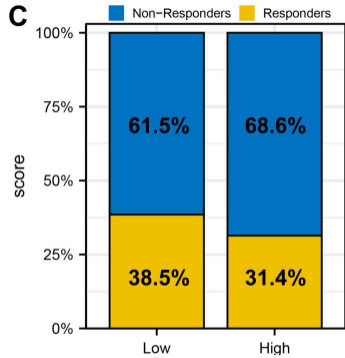

Supplement: Supplementary Materials — Figure S1: the flowchart of whole study. Figure S2: immune correlation analysis of FNDC1 and RSPO3. Notes: A: immune correlation analysis of FNDC1; B: immune correlation analysis of RSPO3. Figure S3: submap analysis was used to indicate patients' sensitivity to PD-1 and CTLA4 therapy. Figure S4: effect of M2 macrophages on GC immunotherapy. Notes: A: correlation of M2 macrophages and TIDE score; B: TIDE score in patients with high and low M2 macrophages infiltration; C: the percentage of immunotherapy responders and nonresponders in patients with high and low M2 macrophages infiltration. [file 1424589.f1.zip › Figure S4 (1).pdf]
